# Evolution of the sex-determining region in *Ginkgo biloba*

**DOI:** 10.1098/rstb.2021.0229

**Published:** 2022-05-09

**Authors:** Wei Gong, Dmitry A. Filatov

**Affiliations:** ^1^ College of Life Sciences, South China Agricultural University, Guangzhou, Guangdong 510642, People's Republic of China; ^2^ Department of Plant Sciences, University of Oxford, South Parks Road, Oxford, UK

**Keywords:** sex chromosomes, *Ginkgo biloba*, genetic mapping

## Abstract

Sex chromosomes or sex-determining regions (SDR) have been discovered in many dioecious plant species, including the iconic ‘living fossil' *Ginkgo biloba*, though the location and size of the SDR in *G. biloba* remain contradictory. Here we resolve these controversies and analyse the evolution of the SDR in this species. Based on transcriptome sequencing data from four genetic crosses we reconstruct male- and female-specific genetic maps and locate the SDR to the middle of chromosome 2. Integration of the genetic maps with the genome sequence reveals that recombination in and around the SDR is suppressed in a region of about 50 Mb in both males and females. However, occasional recombination does occur except a small, less than 5 Mb long region that does not recombine in males. Based on synonymous divergence between homologous X- and Y-linked genes in this region, we infer that the *Ginkgo* SDR is fairly old—at least of Cretaceous origin. The analysis of substitution rates and gene expression reveals only slight Y-degeneration. These results are consistent with findings in other dioecious plants with homomorphic sex chromosomes, where the SDR is typically small and evolves in a region with pre-existing reduced recombination, surrounded by long actively recombining pseudoautosomal regions.

This article is part of the theme issue ‘Sex determination and sex chromosome evolution in land plants’.

## 1. Introduction

Most animal species are comprised of separate male and female individuals, which raises the question: how do separate sexes originate and evolve? Plants provide an excellent opportunity to address this question as separate sexes (dioecy) evolved many times independently and quite recently in different genera [1,2]. Evolution of separate sexes (dioecy) and the diversity of plant mating systems stimulated significant research efforts to understand the drivers of mating system evolution in plants [3–7]. Dioecy is a relatively rare mating system in plants, with only about 5–6% of the species known to be dioecious [1]. On the other hand, dioecy is very common in gymnosperms, where about two thirds of described species (667 of 1033) have separate sexes [8]. The genetic bases of gymnosperm sex determination are not well studied and sex determination in *Ginkgo biloba*, which is the focus of this paper, can serve as a model to start exploring the evolution of mating systems in this interesting and important plant group.

*Ginkgo biloba* is an iconic gymnosperm tree species that is widely cultivated across the world and is praised for its beauty and medicinal properties. This ‘living fossil' is known from fossil data to have existed as a recognizable species at least since the Jurassic [9] and it is a sole representative of Ginkgoales—one of the four extant gymnosperm lineages (cycads, conifers, Ginkgoales and Gnetales). *Ginkgo biloba* is dioecious, with separate male and female individuals, and its sex-determination system is only starting to be explored. Dioecy in *G. biloba* is suggested to have evolved from monoecious ancestors that had ovulate and pollen cones on different parts of the plant [10–12]. Although no fossil data of *Ginkgo* ancestral reproductive structures are available to support this hypothesis, it is in line with evolution of dioecy in conifers where dioecy has evolved from monoecy at least 10 times [12,13].

Despite the large size of the *G. biloba* genome (approx. 10 Gb), it has been sequenced and assembled twice [14,15] and the recent assembly based on long-read sequencing is ‘nearly complete', including 12 large scaffolds corresponding to 12 chromosomes of this species [15]. Based on the previous cytological research, the somatic diploid chromosome number of *G. biloba* is 2*n* = 24 [16–18]. The chromosome complement of *G. biloba* is a bimodal and asymmetrical karyotype [17–19], which is not similar to that reported in any other gymnosperm species [20–22]. The karyotype consists of one pair of long submetacentric chromosomes, one pair of short metacentric chromosomes, and 10 pairs of short subtelocentric chromosomes [16–18].

Though dioecy is widespread in gymnosperm plants (64.6%), only six (0.6%) species from three families are reported to possess sex chromosomes, one of which is *G. biloba* [23,24]. Identification of *G. biloba* sex chromosomes was attempted with optical microscopy and fluorescence *in situ* hybridization approaches but reported karyotypic variation was subsequently found not to be related to sex [17,18,25]. The early works studying sex determination in *G. biloba* reported both XY and ZW sex chromosome systems [17–19,25–28], while recent genome sequencing-based studies indicate that this species has male heterogamety [29,30]. Both of these genome-based studies of the *G. biloba* sex chromosomes reported the sex-determining regions (SDR) on chromosome 2 [29,30]. However, the location and size of the SDR remains contradictory, with one study stating that the SDR is small (4 Mb), including only 16 genes and located between megabases 380 and 384.6 [29], while the other study reported much larger (approx. 27 Mb) SDR including over 200 genes and located between megabases 48 and 75 [30]. Our analysis resolves this controversy and verifies the location of the SDR using an independent set of data.

## 2. Results

### (a) Genetic mapping of the sex locus in *Ginkgo biloba*

To locate the *G. biloba* SDR region we created four genetic families by crossing wild and semi-cultivated trees of this species. These crosses involved three female and four male *G. biloba* individuals (table 1 and the electronic supplementary material, table S1). In addition to that, we sequenced the transcriptome of a male MG4-ZJLKY that was not used in the crosses. As the seedlings from the resulting F_1_ seeds will not reach reproductive age for at least a quarter of a century, their sex was established using a polymerase chain reaction (PCR)-based approach (electronic supplementary material, figure S1) with a sex-specific marker reported previously [30]. The reliability of the PCR-based sex marker was confirmed on nine unrelated females and five unrelated male individuals with sex known from the phenotype (electronic supplementary material, figure S2). In total, this PCR-based approach identified 50 F_1_ females and 51 F_1_ males across the four genetic crosses analysed (electronic supplementary material, table S1).

**Table 1.** Accession information of the male and female *Ginkgo* trees used in the analyses.

code	sex^a^	location	voucher	latitude	longitude
FG1-NXDLB	♀	Dalingbei, Pingtian, Nanxiong, Guangdong	ZH010	25.22° N	114.63° E
FG2-NXLBT2	♀	Lingbeitang, Pingtian, Nanxiong, Guangdong	ZH011	25.22° N	114.63° E
FG3-NXLBT3	♀	Lingbeitang, Pingtian, Nanxiong, Guangdong	ZH012	25.22° N	114.63° E
MG1-NXAB	♂	Aobei, Pingtian, Nanxiong, Guangdong	ZH013	25.22° N	114.63° E
MG2-NXDLB	♂	Dalingbei, Pingtian, Nanxiong, Guangdong	ZH009	25.22° N	114.63° E
MG3-NXPH	♂	Pinghu, Pingtian, Nanxiong, Guangdong	ZH014	25.22° N	114.63° E
MG4-ZJLKY^b^	♂	Zhejiang Forestry Inst., Hangzhou, Zhejiang	ZH019	30.22° N	120.03° E
MG5-ZJTM	♂	Tianmu Mountain, Lian'an, Zhejiang	ZH020	30.35° N	119.43° E

^a^The sex of the individuals was identified by phenotypic observation.

^b^Male MG4-ZJLKY was not used in the crosses, but it was used in other analyses.

In order to obtain genotypic information for coding regions of the *G. biloba* genome we used Illumina paired-end transcriptome sequencing for parents and F_1_ progeny of the four crosses, which in total yielded 379.26 Gb of sequence data across the adult plants and 101 F_1_ individuals (electronic supplementary material, table S1). These sequence reads were mapped to coding regions (CDS) annotated in the female reference genome [15], and single nucleotide polymorphisms (SNPs) were called and filtered as described in the methods.

Given the limited size of individual families (19 to 30 F_1_-individuals per cross; electronic supplementary material, table S1), we included all four families in a joint analysis to increase resolution of the genetic map. The genetic map was reconstructed with the lepMap3 software [31]. The total length of the resulting female and male maps were 1173 cM and 1225 cM, respectively, and included 12 linkage groups (electronic supplementary material, table S2), which corresponds to the number of chromosomes in this species. The maps were based on 10 016 SNP markers in 4915 protein coding genes. The maps for individual linkage groups included 409 to 1262 SNPs in 206 to 594 genes (electronic supplementary material, table S2). The length of linkage groups ranged from 33 to 122 cM in the female map and from 47 to 143 cM in the male map.

**Figure 1.**
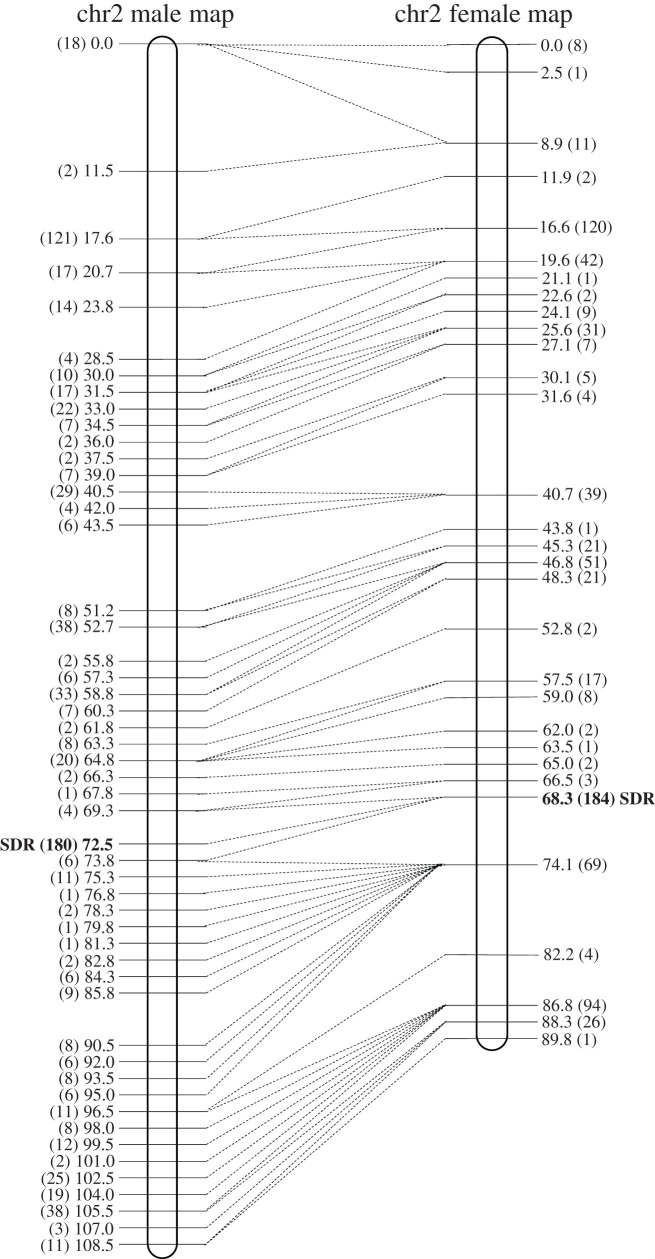
Male and female genetic maps for chromosome 2. Genetic positions (cM) are shown to the left of the male map and to the right of the female map. The numbers in brackets show how many markers co-locate to the same position in the map. The locations of the markers in the male and female maps are connected with a dashed line.

The sex locus is mapped to the central part of chromosome 2. The map of chromosome 2 is based on 789 markers in 392 genes. The female and male maps for this chromosome were 90 and 109 cM long, respectively (figure 1). In the male map, the sex locus co-locates with 180 markers in 33 genes that all have the same genetic position (72.53 cM). In the female map, it co-locates with 184 markers in 34 genes at genetic position 68.34 cM (figure 1; electronic supplementary material, table S2). Co-location of multiple markers indicates that recombination is suppressed or reduced in the region around the sex locus in both male and female maps. Recombination suppression in the region around the sex locus was confirmed once the genetic maps were integrated with the genome sequence [15].

### (b) Integration of the genetic map and the genome sequence

The combined analysis of the genetic map and the female reference genome [15] revealed that the order of genes is mostly colinear for all chromosomes, except chromosomes 1 and 4 (electronic supplementary material, figure S3). According to the genetic map, the first approximately 400 Mb of the scaffold for chromosome 1 is misplaced in the genome assembly—this region should either be on the other side of chromosome 1 or it may belong to a different chromosome, as recombination distance between markers in this region and the rest of the chromosome 1 is over 90 cM (electronic supplementary material, figure S3). A similar problem is present on chromosome 4: the genetic distance between multiple markers in the first 200 Mb and the rest of chromosome 4 is over 90 cM (electronic supplementary material, figure S3), indicating that the first approximately 200 Mb of chromosome 4 should be moved to the other side of chromosome 4 or disconnected from this chromosomal scaffold. As fixing errors in the *G. biloba* genome sequence assembly is beyond the scope of the current paper, we flag these likely errors in the female genome reference [15] and leave their resolution to later studies.

**Figure 2.**
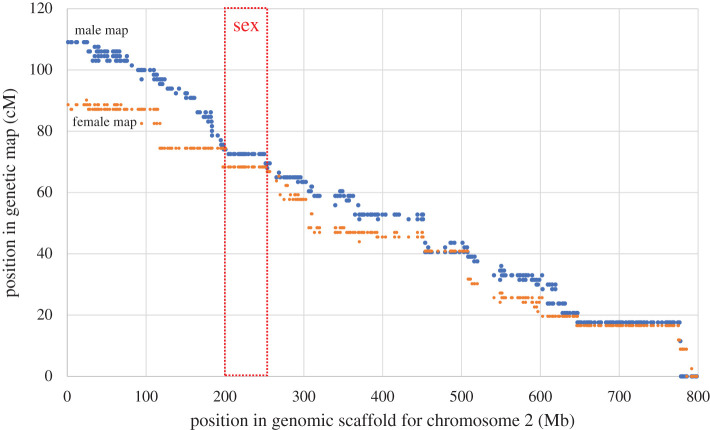
Genetic versus physical positions on chromosome 2 in the female *G. biloba* genome assembly [15] for male (blue) and female (orange) genetic maps. The dotted box shows the genomic location of the sex-determining locus, as located by genetic mapping. For other chromosomes see the electronic supplementary material, figure S3. (Online version in colour.)

Focusing on chromosome 2 and the SDR, the integrated map and female genome sequence [15] revealed that the region with reduced recombination surrounding the sex locus is approximately 50 Mb long (figure 2). It is flanked by genes *evm.chr2.722* and *evm.chr2.564* (electronic supplementary material, table S3) located at 251.7 and 200.8 Mb on chromosome 2 in the female *G. biloba* genome reference assembly [15]. According to the annotation of the reference sequence, this region contains 159 protein-coding genes, however, we were able to genetically map only 34 of these genes in the genetic map, with other genes probably missing informative markers owing to lack of polymorphic sites and/or low expression in our transcriptome sequence-based genotyping analysis.

### (c) The size and age of the non-recombining Y-specific region

The size of the *G. biloba* non-recombing region surrounding the sex locus, obtained from our genetic mapping data is likely an overestimate, as rare recombination events in this region would not be detected with the relatively small number of F1 progeny analysed. To locate the non-recombining Y-specific region (NRY) boundaries more accurately we looked for SNPs that segregated as Y-linked in independent genetic crosses. As the males listed in table 1 are unrelated, the SNPs that are Y-specific in all the crosses are likely to be located in the NRY. Truly Y-linked SNPs are expected to never be found in females, while the SNPs closely linked to the NRY could be separated from the NRY by recombination and occasionally would be found in females. Our analysis of four genetic crosses identified 61 putative NRY SNPs in four adjacent genes (*evm.chr2.642* to *evm.chr2.645*; electronic supplementary material, table S3) located between 222.5 Mb and 223.9 Mb on the chromosome 2 genomic scaffold in the latest genome assembly [15]. None of these SNPs were present in any of the female F_1_ or female parents analysed, which is consistent with location of these genes and SNPs in *G. biloba* NRY. It is worth noting that all four of these genes are actively expressed in both sexes (see below), so the presence of Y-SNPs only in males is not owing to male-specific expression of these genes.

The age of *Ginkgo* NRY could be measured from synonymous sequence divergence between the X- and Y-linked gametologues. Prior to NRY formation, recombination should have precluded accumulation of differences between the alleles, so the divergence between the homologous X- and Y-linked genes owing to neutral mutations (e.g. at synonymous codon positions) can serve as the clock measuring the time since recombination cessation. To measure this divergence, we reconstructed the sequences of Y-alleles for the X-linked genes located in the *G. biloba* sex-determining region. As described in the methods, this reconstruction is based on the identification of Y-specific sequence reads containing the Y-linked (male-specific) SNPs. That is why this analysis was done only for the four NRY genes described above, where such Y-SNPs were found.

For all NRY genes, the length of the reconstructed Y-linked sequence covered 100% of the length of the CDS of X-linked gametologues. The reconstructed Y-sequences were nearly identical to, but longer than the Y-sequences reconstructed for these genes by [30], see the electronic supplementary material, table S3 (sequences *MSTRG.230.1.p1, MSTRG.108.1.p1, MSTRG.304.2.p1* and *MSTRG.187.1.p1*, which were obtained from [30] by request). The reconstruction of the sequence for Y-linked gametologues allowed us to analyse X:Y divergence based on 80.33 to 622.75 synonymous sites per gene (table 2). Synonymous X:Y divergence (*K_s_*) ranged from 1.8% for gene *evm.chr2.645* to 10.2% for gene *evm.chr2.642* (table 2). An estimate of the NRY age depends on the mutation rate and average generation time, neither of which are known for *G. biloba*. Given that *G. biloba* does not start reproducing until it is at least 25 years old, we can use *g* ∼ 25 years as a minimal estimate of generation time. Gymnosperms have been reported to have a slower evolutionary rate compared to angiosperms [32], so we can assume that *m* ∼ 10^−8^ is a maximal estimate of the per nucleotide per generation mutation rate, which allows us to calculate the minimal age of the *G. biloba* NRY as *T* = *g K_s_*/2 *m* = 25*0.1/2*10^−8^ = 125 million years. We use the gene with *K*_s_ = 0.1 (for gene *evm.chr2.642*) in this calculation because the genes with lower *K*_s_ may have been added to NRY more recently, so their *K*_s_ may not reflect the age of the NRY. Alternatively, the variation in *K*_s_ among the NRY genes may reflect local variation in mutation rate, in which case it would be more appropriate to use the average *K*_s_ = 0.048 across all four NRY genes. This gives the minimal age of the *G. biloba* NRY as *T* = *g K*_s_/2 *m* = 25*0.048/2*10^−8^ = 60 million years.

**Table 2.** Synonymous and non-synonymous divergence between the X- and Y-linked gametologs in the *G. biloba* non-recombining Y-specific region.

gene	CDS length	SynDif^a^	SynPos^b^	*K*_s_^c^	NSynDif^d^	NSynPos^e^	*K*_a_^f^	*K*_a_/*K*_s_
*evm.chr2.642*	1932	43.50	455.25	0.102	44.50	1476.75	0.031	0.30
*evm.chr2.643*	331	3.00	80.33	0.038	5.00	249.67	0.020	0.53
*evm.chr2.644*	1230	9.00	275.75	0.033	8.00	954.25	0.008	0.25
*evm.chr2.645*	2760	11.00	622.75	0.018	14.00	2137.25	0.007	0.37

^a^Number of synonymous differences.

^b^Number of synonymous positions analysed.

^c^Synonymous divergence.

^d^Number of non-synonymous differences.

^e^Number of non-synonymous positions analysed.

^f^Non-synonymous divergence.

### (d) Do *Ginkgo biloba* non-recombining Y-specific region genes undergo degeneration?

In the absence of recombination, the efficacy of natural selection to eliminate deleterious mutations and fix advantageous mutations is reduced, which is expected to lead to genetic degeneration of genes in the non-recombining regions, such as is found in the non-recombining regions of the Y(or W)-chromosomes of many organisms [33,34].

**Figure 3.**
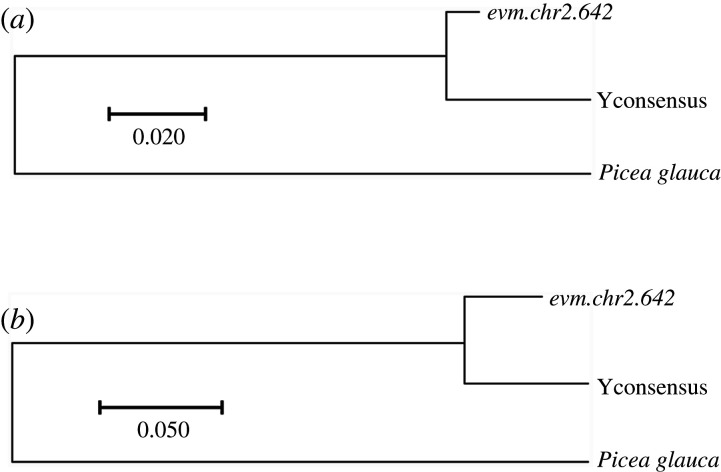
Maximum-likelihood phylogeny for the 1st (*a*) and 3rd (*b*) codon positions in the gene *evm.chr2.642*.

Elevated substitution rate at non-synonymous positions is one of the signs of inefficient purifying selection and ongoing degeneration. In all the NRY genes analysed, non-synonymous divergence between the X- and Y-gametologues (*K*_a_) was lower than synonymous divergence (*K*_s_) (table 2). The *K*_a_/*K*_s_ ratio ranged from 0.25 to 0.53, indicating the presence of relatively weak purifying selection eliminating deleterious mutations at non-synonymous sites of these sex-linked genes. To test whether the Y-linked alleles accumulate more non-synonymous substitutions compared to the corresponding X-linked alleles, as expected for genes in the non-recombining regions [33,34], we used homologous genes from an outgroup *Picea glauca* [35] to ‘polarize’ the pairwise X : Y divergence (figure 3), identifying the substitutions that occurred in the X- or Y-linked copies of the sex-linked genes. Tajima's relative rates test [36] revealed a significant excess of substitutions on the Y compared to the X at the 1st codon position of the *evm.chr2.642* gene (table 3 and figure 3). All other comparisons showed no significant difference in the number of substitutions on the X- and the Y-chromosomes.

**Table 3.** Tajima's relative rates test [36] comparing substitutions in the X- and Y-linked gametologues.

gene:	*evm.chr2.642*			*evm.chr2.643*			*evm.chr2.644*			*evm chr2.645*		
codon pos:	1st	2nd	3rd	1st	2nd	3rd	1st	2nd	3rd	1^st^	2nd	3rd
invariable:	515	574	408	100	100	79	317	261	298	198	208	170
substitutions:												
in X-allele	4	6	17	0	1	2	2	3	0	1	1	0
in Y-allele	14*	6	23	2	2	1	2	6	2	0	1	0
in outgroup	100	49	179	5	4	25	34	86	55	56	45	84
chi^2^	**5.56**	0.00	0.90	2.00	0.33	0.33	0.00	0.14	2.00	1.00	0.00	0.00
* p*	**0.018**	1.00	0.34	0.16	0.56	0.56	1.00	0.71	0.16	0.32	1.00	1.00

Reduced expression of the Y- compared to the X-linked gametologues is another sign of ongoing genetic degeneration of Y-linked genes. The analysis of expression of sex-linked genes in males and females revealed that, as expected, expression of Y-alleles is not detectable in females, indicating that the expression of homologous X- and Y-linked genes are accurately distinguished in our expression analysis. Furthermore, comparing expression between the sexes, the X-linked genes in males were expressed at about twice the lower level compared to females (table 4), as expected from the number of copies of X-linked genes present in the two sexes, which is also consistent with accurate quantification of gene expression despite low divergence between homologous X- and Y-linked genes. In three out of four NRY genes in males, expression of X- and Y-linked genes was nearly identical (table 4). On the other hand, expression of the Y-linked copy of the *evm.chr2.643* gene was consistently (across the families) lower compared to its X-linked gametologues in males (table 4), suggesting partial degeneration or silencing of this gene on the Y-chromosome. No evidence of elevated expression of the X-linked copy of the *evm.chr2.643* gene to compensate for reduced expression of the Y-linked copy in males was found.

**Table 4.** Gene expression in four *G. biloba* genes on the X-chromosome in males (mX) and females (fXX) and the Y-chromosome in males (mY), shown separately for each of the genetic crosses (PH, DLB, LTM and LAB).

family (sample size)				median FPKMs		
gene_id	mY/mX	mXY/fXX	mX/fXX	fXX	mX	mY
**PH (11F, 9M)**						
*evm.chr2.642*	1.077	1.035	0.499	40.92	20.40	21.97
*evm.chr2.643*	0.829	0.783	0.428	32.99	14.13	11.71
*evm.chr2.644*	1.061	1.060	0.514	29.22	15.03	15.94
*evm.chr2.645*	1.246	0.995	0.509	15.75	8.01	9.98
**DLB (12F, 14M)**						
*evm.chr2.642*	1.301	1.141	0.496	33.68	16.70	21.72
*evm.chr2.643*	0.522	0.850	0.559	65.62	36.66	19.14
*evm.chr2.644*	1.076	1.149	0.554	24.89	13.78	14.83
*evm.chr2.645*	1.196	1.164	0.616	12.93	7.97	9.53
**LTM (11F, 13M)**						
*evm.chr2.642*	1.342	1.397	0.596	34.14	20.36	27.33
*evm.chr2.643*	0.620	0.620	0.383	54.50	20.86	12.94
*evm.chr2.644*	0.975	0.892	0.452	30.56	13.80	13.46
*evm.chr2.645*	1.146	0.988	0.536	14.58	7.82	8.96
**LAB (10F, 13M)**						
*evm.chr2.642*	1.087	0.971	0.465	44.91	20.90	22.71
*evm.chr2.643*	0.492	0.990	0.663	30.37	20.15	9.92
*evm.chr2.644*	1.168	0.926	0.427	31.42	13.43	15.68
*evm.chr2.645*	1.382	1.083	0.530	15.06	7.98	11.03

### (e) The comparison of sex-linked genes between the studies

**Figure 4.**
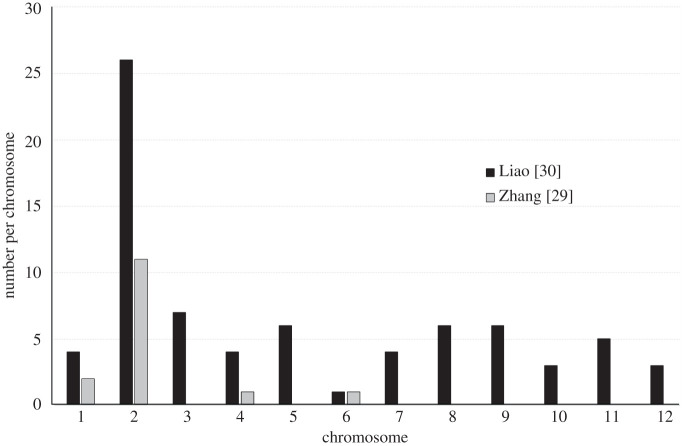
The distribution of best blast hits for putatively sex-linked genes from [29,30] across the 12 chromosomes in the latest *G. biloba* female genome assembly [15].

The comparison between the studies reveals that our four NRY genes represent reciprocal best blast hits with four out of more than 200 putatively sex-linked (PSL) genes reported by [30] and three of our NRY genes correspond to three of the 16 PSL genes reported by [29]. Furthermore, eight and 12 of the PSL genes reported by [29] and [30], respectively, represent reciprocal best blast hits with some of the genes in the approximately 50 Mb region around the NRY that showed no recombination in the genetic crosses described above (electronic supplementary material, table S3). PSL genes reported in the previous studies [29,30] have reciprocal best blast hits with genes on different chromosomes in the latest female genome assembly [15], though most PSL genes do show close homology with the genes on chromosome 2 (figure 4).

It is worth noting that the putative *G. biloba* sex-determining gene *GbMADS18* (Genbank accession MN548764.1) reported by [30] shows close homology (96.4% sequence identity) with gene *evm.chr2.641* that is adjacent to our four NRY genes, though the region of homology is quite short—only 137 bases. The absence of a complete *GbMADS18* gene in the female genome reference [15] is probably because the gene is Y-specific and present only in male genomes. No expression of the *GbMADS18* or *evm.chr2.641* genes was detected, hence they could not be used in our transcriptome-based analysis. NRY may include *evm.chr2.641* and a few other adjacent genes that could not be tested in our analysis owing to lack of expression. However, NRY is unlikely to be larger than 5 Mb and extend beyond *evm.chr2.638* and *evm.chr2.649* genes located at 220.99 Mb and 225.22 Mb of chromosome 2, respectively, because no Y-linked SNPs were found in these genes or any of the genes located at positions less than 220 Mb and greater than 225 Mb on the chromosome 2 (table 5; electronic supplementary material, table S3).

**Table 5.** The comparison of SDR genes between the studies. The genes with male-specific Y-SNPs are highlighted in bold.

gene	Pos. (Mb)	Y-SNPs	Zhang [29]	Liao [30]	putative function
*chr2.630*	218.69	0			nucleolar GTP-binding protein 1-like
*chr2.631*	218.91				LURP-one-related 12-like protein
*chr2.632*	218.91				LURP-one-related 6-like protein
*chr2.633*	219.24				two-component response regulator-like PRR73
*chr2.634*	219.66				no homology
*chr2.635*	220.06			*MSTRG.35.1.p1*	putative disease resistance protein
*chr2.636*	220.18	0	*Gb_30344 (S3)*	*MSTRG.183.1.p2*	FGGY family of carbohydrate kinases
*chr2.637*	220.69	0		*MSTRG.399.2.p1*	adenosylmethionine-8-amino-7-oxononanoate aminotransferase
*chr2.638*	220.99	0	*Gb_30343 (S3)*		DAPA aminotransferase [*Arabidopsis thaliana*]
*chr2.639*	221.46		*Gb_28587 (S2)*		MADS-box transcription factor GbMADS9
*chr2.640*	221.71			*MSTRG.305.1.p1*	MADS-box transcription factor GbMADS4
*chr2.641*	222.14			*MSTRG.480.1.p1*	MADS-box transcription factor GbMADS10
***chr2.642***	222.55	**32**	*Gb_28585 (S2)*	*MSTRG.230.1.p1*	protein NUCLEAR FUSION DEFECTIVE 4-like
***chr2.643***	222.97	**5**	*Gb_28589 (S2)*	*MSTRG.108.1.p1*	alpha/beta hydrolase domain-containing protein 17B-like
***chr2.644***	223.53	**11**		*MSTRG.304.2.p1*	unknown protein
***chr2.645***	223.87	**13**	*Gb_15883 (S1)*	*MSTRG.187.1.p1*	two-component response regulator ORR24-like protein
*chr2.646*	224.37		*Gb_15885 (S1)*		lysine-specific demethylase JMJ706-like
*chr2.647*	224.49				no homology
*chr2.648*	224.88		*Gb_15886 (S1)*		brassinosteroid-related acyltransferase 1
*chr2.649*	225.1	0			pentatricopeptide repeat-containing protein At5g10690
*chr2.650*	225.42	0			calmodulin-binding protein 60C

## 3. Discussion

In this paper, we focused on genetic mapping of the SDR in *G. biloba* using a set of four independent genetic crosses. This allowed us to build separate female and male genetic maps, integrating information across the genetic crosses. Overall, the male and female genetic maps are similar, but the male map tends to be slightly longer for most chromosomes, which is consistent with slight male bias in heterochiasmy in gymnosperms (e.g. fig. 1 in [37]). But the length difference between the male and female maps is minor and the rate and distribution of recombination in meiosis do not show large differences between the two sexes. This contrasts with many other species with fairly strong sex differences in overall recombination rate and distribution of crossovers along the chromosomes [38].

The use of SNPs in expressed genes as genetic markers allowed us to integrate the maps with the female genome sequence [15] and to measure the recombination rate in different genomic regions. This revealed several relatively short regions where recombination is reduced in both sexes, with no obvious pattern in the location of such regions in the chromosomes. In particular, we detected no evidence for large regions with reduced recombination in the middle of the chromosomes (presumably in pericentromeric regions), as reported for other plant species [39]. The standard karyotype of *G. biloba* is composed of 12 chromosome pairs with metacentric, submetacentric and subtelocentric chromosomes [17–19,25]. Thus, the lack of extended regions with low recombination in central parts of the chromosomes suggests that pericentromeric suppression of recombination is not extensive in *G. biloba*.

Our genetic mapping of the SDR located it to the central part of chromosome 2. The SDR locates to a region approximately 50 Mb long where recombination is rare or absent in both males and females. This situation contrasts with ‘typical' sex chromosomes found in many animals (e.g. mammals, birds) and some plants (*Silene latifolia* [40], *Rumex hastatulus* [41]) where recombination is suppressed only on the sex-specific Y(or W)-chromosome, while the other sex chromosome X(or Z) recombines normally in the homogametic sex. Low recombination around the *G. biloba* SDR in both sexes suggests that in this species sex-determining gene(s) evolved in a region with pre-existing low recombination, as was also hypothesized for papaya [42]. Alternatively, recombination suppression around the SDR may have evolved after the rise of the sex locus in this region, as postulated by the classic 2-locus model of sex chromosome evolution [7], though, in the case of *G. biloba*, recombination suppression evolved in a non-sex-specific manner. In the absence of other *Ginkgo* species with different mating systems and/or sex chromosomes, it is difficult to test these hypotheses.

The location of the SDR on chromosome 2 is consistent with other studies [29,30], though the exact position of the SDR on that chromosome was contradictory. In particular, [29] reported that the SDR is located at 380.00 Mb–384.60 Mb on chromosome 2, while according to [30], it is located at 48 Mb–75 Mb on chromosome 2. This contradiction is partly owing to the use of different genomic references in these studies. The former study used the draft female genome assembly published by [14], while the latter used an unpublished female assembly reported in the form of a PhD thesis [43] and unfortunately that genome assembly is not publicly available. It is possible that the unpublished assembly of [43], which was used as a reference sequence by [30], is the same as the latest ‘nearly complete' publicly available *G. biloba* female genome assembly [15], though this is not clear from the papers. The location of the SDR around position 222 Mb in the latest assembly [15] contradicts the SDR location at 48 Mb–75 Mb on chromosome 2 reported by [30], suggesting that the assembly used by [43] and by [30] is different from the genome that is publicly available [15]. This makes it difficult to verify the results of [30] and work out the reasons for the discrepancies in reported SDR location between the papers.

On the X-chromosome, the SDR appears to comprise a fairly small (less than 5 Mb long) region, where recombination is entirely suppressed in males. The size of the corresponding NRY region is less clear. In our analysis, we focused on the genes present in the SDR region on the X-chromosome (in the genome assembly for a female *G. biloba* [15]) and their homologous on the Y-chromosome. Thus, any Y-specific genes that are not present on the X were missed in our analysis. Owing to the accumulation of repetitive DNA, and translocation of genes from other chromosomes to the Y-chromosome, as reported for other species (e.g. [44]), the NRY may be larger than the corresponding SDR region on the X-chromosome, which may partly account for the larger estimate of SDR size reported by [30], as that study reconstructed the Y-linked contigs based on male-specific k-mers. The highly dynamic nature of repetitive DNA in the NRY, with frequent inversions, gains and losses of genetic material owing to recombination between different copies, as described in other species [45], may also affect the NRY in *G. biloba*. This may cause the NRY region to vary in size among males of the same species, which could, at least partly, account for the controversy with cytogenetic reports of hetero- and homo-morphic sex chromosomes in *G. biloba* [17,18,25].

The fairly large size of the SDR (more than 200 genes) reported by [30] may be explained by the fact that they used a single sibling family in their analysis, which limited the number of recombination events detectable in the family of relatively small size (100 individuals). Given the approximately 50 Mb low-recombination region we detected around the SDR, all the genes in that region may appear completely sex-linked in a sibling family of limited size, yet, most of that region may not be fully sex-linked and occasionally recombine, which can be detected only by the analysis of multiple unrelated individuals, or using sibling families of very large size. As such, the more than 200 SDR genes reported by [30] are likely to be an overestimate of the actual size of the fully sex-linked region in *G. biloba*. It is more difficult to explain why reciprocal best blast hits for many of the putatively sex-linked genes identified by [30] are located on other chromosomes (figure 4), according to the latest genome assembly [15]. It is possible that the NRY is larger and contains many more genes than the corresponding region on the X-chromosome, including the genes transferred to the Y from other chromosomes. This may, at least partly, account for the homology of many sex-linked genes reported by [30] to genes on other chromosomes.

The age of the *G. biloba* SDR, as inferred from our analysis of synonymous divergence for X- and Y-linked gametologues, is comparable to ancient sex chromosomes in mammals [46,47] and birds [48], and it is older than in many other plant species analysed, such as *Silene* [49], *Rumex* [50], papaya [42], *Asparagus* [51]. Our estimate of the *G. biloba* SDR age contrasts with the much lower estimate of approximately 14 Myr reported by Zhang *et al.* [29]. The estimate of the SDR age in *G. biloba* critically depends on the assumptions of the per-base per-generation mutation rate and generation time, which are not known for this species. The higher mutation rate and shorter generation time would lead to younger estimates of the SDR age and it is not clear what values for these parameters were used by Zhang *et al.* [29] to estimate the age of SDR. We used a minimal estimate of the generation time (25 years) and a fairly high ad hoc mutation rate (10^−8^ mutations per site per generation) to obtain the minimal age of the non-recombining region on the Y-chromosome (approx. 125 million years). Given that *G. biloba* can live for hundreds of years and it does not start reproducing until it is at least 25 years old, its average generation time is probably much higher than 25 years. Based on the divergence between pine and spruce at synonymous sites of 3723 orthologous, it was reported that the substitution rate in conifers is approximately 0.68 × 10^−9^ per synonymous site per year [32]. Assuming this substitution rate for *Ginkgo*, we get an estimate *T* = *K*_s_/2 *m* = 0.1/0.68*10^−9^∼147 million years for the age of *G. biloba* sex chromosomes. However, this is also likely to be an underestimate because [32] estimated the substitution rate for fast-growing pine and spruce, while slow-growing *G. biloba* is likely to have a longer generation time and hence a slower per-year substitution rate assuming the same per-generation mutation rate.

The limited number of genes with X- and Y-linked sequences available to calculate X:Y divergence, and variation of synonymous X:Y divergence among the genes (table 2) present additional difficulties for estimation of the SDR age in *G. biloba*. To obtain a conservative minimal estimate for the SDR age one can average synonymous X:Y divergence across the four NRY genes, which gives *K*_s_ = 0.048, placing the minimal estimate for the sex chromosome age in *Ginkgo* at the Cretaceous–Palaeogene boundary, assuming 25 years as generation time and 10^−8^ as per-nucleotide per generation mutation rate. It is difficult to reliably estimate the age of *G. biloba* sex chromosomes based on so few genes, but it is clear that the *Ginkgo* SDR is at least of Cretaceous origin. This conclusion fits well with the palaeontological evidence: in the 121 Myr old fossil, ovulate organs of *Ginkgo* are similar to those of the present-day *G. biloba*, while the *Ginkgo* plants from the Jurassic appear to have a more primitive type of reproductive organs [9,11]. Furthermore, the depth of the X:Y clade relative to the total depth of the phylogeny (figure 3) is also consistent with Cretaceous origin of the *Ginkgo* SDR, assuming the divergence of Ginkgoales from other gymnosperms back in the Palaeozoic [11]. As such, the SDR age is probably older than the age of the species *G. biloba* that is thought to have originated less than 50 Ma [9].

Our conclusion that *Ginkgo* sex chromosomes are at least of Cretaceous origin goes against the claim of [29] that the SDR originated in the last 14 million years. However, the authors of that paper reconstructed Y-linked alleles only for the genes with low X:Y divergence in the areas of the SDR they called S1 and S3. Y-alleles in the older part of the SDR (called S2 in [29]) were not reconstructed, so they could not estimate the age for the oldest part of the sex chromosomes. Two (*evm.chr2.642* and *evm.chr2.643*) out of four genes with Y-linked SNPs we used for reconstruction of Y-alleles (table 2) are located in the older S2 area of the SDR (table 5). These two genes do show the highest X:Y divergence (table 2), leading to older SDR age inferred in our study.

The variation in synonymous X:Y divergence across the genes (table 2) may reflect an expansion of the NRY over time. The order of genes on the genomic scaffold from *evm.chr2.642* having the highest X:Y divergence (*K*_s_ = 0.1) to *evm.chr2.645* having the lowest divergence (*K*_s_ = 0.02) is consistent with such expansion. The model of NRY expansion over time seemingly contradicts our observation that recombination around the SDR in *G. biloba* is reduced in both sexes. However, the recombination suppression in the approximately 50 Mb region around the SDR is incomplete, as indicated by the lack of Y-specific SNPs outside the small NRY region. Thus, pre-existing reduced recombination in the approximately 50 Mb region around the SDR does not contradict the gradual expansion of the small NRY region where recombination is suppressed completely.

Our analysis detected little evidence for genetic degeneration of Y-linked genes. A slight reduction of the Y- compared to X-expression for gene *evm.chr2.643*, and significantly faster non-synonymous substitution rate in the Y- compared to X-linked gametologues of the *evm.chr2.642* gene, were the only signs of ongoing Y-degeneration. This may seem surprising given our conclusion that the *Ginkgo* SDR is fairly ancient [52]. However, the rate of genetic degeneration depends on the number of genes linked together in the NRY. In particular, with relatively few functional genes left on the old Y(or W)-chromosomes in mammals and birds the rate of degeneration is expected to be very slow [53,54]. Thus, in small NRYs, such as the one found in *G. biloba*, genes are not expected to undergo rapid degeneration. Furthermore, the long generation time of *G. biloba* and its (likely) low mutation rate, result in a fairly slow rate of molecular evolution, which is also expected to slow down the process of genetic degeneration.

## 4. Conclusion

Our paper resolves the ambiguities and contradictions present in the literature [29,30] regarding the location of the sex-determining region in *G. biloba* genome. The results of our analyses indicate that the SDR is located in the central part (around megabase 222) of chromosome 2 in the latest ‘nearly complete' assembly of the female *G. biloba* genome [15]. The region of chromosome 2 (the X) around the sex-determining region where recombination in males is suppressed completely is fairly small—it is at least 1.3 Mb and probably less than 5 Mb long. The size of the corresponding NRY region is unclear, but may be larger owing to accumulation of repetitive DNA, which typically occurs in non-recombining regions. The small SDR is embedded into an approximately 50 Mb long genomic region where recombination is reduced in both sexes, but does occur occasionally, which prevents the divergence between the X- and Y-linked alleles of the genes in this region. The NRY appears to have expanded over time, though these expansion(s) were small, adding only a few genes to the NRY. The divergence between the X- and Y-linked gametologues is modest (*K*_s_ less than 10%), which prompted the previous study to claim that *G. biloba* sex chromosomes are of ‘recent origin' [29]. However, our analysis shows that *Ginkgo* sex chromosomes are at least of Cretaceous origin and the relatively low rate of synonymous X:Y divergence reflects the slow evolution of this species owing to long generation time and (likely) low mutation rate.

## 5. Methods

### (a) Genetic crosses

Three female and four male trees of *G. biloba* were selected as candidates for genetic crosses. Accession information is listed in the electronic supplementary material, table S1. Artificial pollinations were conducted in the county of Pingtian, Nanxiong, Guangdong Province, in southern China. Large population size and numerous old trees of *G. biloba* have been identified and documented in Nanxiong, which is thought to be one of the glacial refugia of *Ginkgo* as revealed by genetic diversity analysis [55]. To ensure a high seed rate, we chose three female *Ginkgo* trees which are recorded to possess high seed production and documented to have an average age of more than 100 years. Three male *Ginkgo* trees were also selected in the same county of Pingtian. They are recorded as wild trees over 1000 years old, having a diameter at breast height of more than 80 cm and a height of approximately 20 m. One other male *Ginkgo* tree was chosen from Tianmu Mountain, Lin'an, Zhejiang Province, in eastern China. This tree is also growing in a refugium population of *Ginkgo*. In April, 2016, the branches of the female trees were wrapped with parchment paper and the male flowers from the nearby male trees were all removed before flowering to avoid any pollen pollution. Artificial pollinations were conducted for four crosses: LAB (MG1_NXAB × FG3_NXLBT3), DLB (MG2_NXDLB × FG1_NXDLB), PH (MG3_NXPH × FG2_NXLBT2) and LTM (MG5_ZJTM × FG3_NXLBT3). For each cross, more than 100 seeds were collected and germinated. In total, 101 *Ginkgo* F_1_ seedlings were grown in the greenhouse. In the spring of 2017, leaves were collected and total RNA extracted using RNA Easy Fast (DP452) according to the manufacturer's instructions (Tiangen Biotech (Beijing) Co., Ltd.).

### (b) Sex identification of F_1_ progeny

We used the primer pairs of the Y-linked gene *GbMADS18* reported by [30] to identify the sex of the F_1_ progeny. The male and female parents and several other *Ginkgo* trees with known sex were also tested as controls for accurate sex identification. The PCR-based marker produced a 1030 bp fragment precisely and robustly only in male leaf samples, while only positive control bands were amplified in female leaf samples. The PCR reactions were carried out in a total volume of 25 µl, containing 12 ng of template DNA, 1.25 units of Taq DNA polymerase, 0.8 µM of forward and reverse primer, 0.2 µM of dNTP, 2 mM of MgCl_2_, 2.5 µl of 10× PCR buffer and 14.25 µl of sterilized double-distilled water. Thermo cycling conditions were: initial denaturation at 94°C for 5 min; 32 cycles of denaturation at 94°C for 30 s, annealing at 57°C for 30 s and polymerization at 72°C for 45 s; and a final elongation step at 72°C for 5 min. The amplified fragments were visualized via gel documentation Tanon 3500. The upper band shows the positive control and the lower band is the Y-specific marker (electronic supplementary material, figures S1, S2).

### (c) Transcriptome sequencing and data processing

Paired-end Illumina transcriptome sequencing was conducted by Novogene Co. Ltd. for parents and F_1_ progeny of the four crosses. All sequence reads were submitted to the SRA database under project number PRJNA758727. For the complete list of samples and accession numbers see the electronic supplementary material, table S1.

Quality checking of the sequence reads was done with *FastQC* v. 0.11 (available from http://www.bioinformatics.babraham.ac.uk/projects/fastqc). *CutAdapt* v. 1.4 [56] and *Trimmomatic* v. 0.32 [57] were used to remove Illumina adapters and to trim low-quality bases at the ends of the reads. Trimming was done for leading and trailing bases with quality below 5, as well as for bases with the average quality less than 15 per 4 consecutive bases. Reads shorter than 36 bases after trimming were discarded.

For expression analysis, RNA-seq reads were mapped to the CDS sequences from the reference genome [15] and gene expression measured using *RSEM* v. 1.3.3 [58] with default parameters. As shown in our previous work, this approach is sufficiently accurate to distinguish the expression of X- and Y-linked gametologues with divergence greater than 1% [59].

For SNP calling, RNA-seq reads were mapped to the CDS sequences extracted from the reference genome [15], with *bwa-mem* v. 0.7.17-r1188 [60]. Duplicated reads were removed with *Picard* v. 2.26 (https://broadinstitute.github.io/picard/). The genome analyses toolkit (*GATK*) v. 4.1.2.0 [61] was then used for base quality recalibration, local realignment around insertions/deletions. The resulting bam files were sorted, indexed and used for SNP calling with *samtools* v. 1.7 [62]. The resulting multi-sample vcf file with SNP calls for parents and F_1_ progeny of the four crosses was used for reconstruction of the genetic map and location of the sex-determining region.

### (d) Construction of genetic maps

For genetic mapping we used *lepMap3* v. 0.2 [31]. Before construction of genetic maps, the relatedness of parents and progeny was checked with the identity by descent (*IBD*) tool in *lepMap3* [31]. For this purpose, we used a subset of 5000 SNPs selected randomly across the genome. As expected, most F_1_ individuals had approximately 0.5 identity to each of the parents, while five F_1_ individuals in the PH cross (PH203, PH208, PH221, PH224 and PH232) had lower *IBD* (less than 0.35) with their presumed father (MG3) and were excluded from analyses as possible contaminants. The remaining individuals were used to call the parental genotypes with the *ParentCall2* module of *lepMap3*, including parameter halfSibs = 1 to take into account the half-sibling family structure. SNP filtering was done with *Filtering2* module of the *lepMap3*, with parameter dataTolerance = 0.001, to exclude the markers showing distorted segregation. The resulting filtered SNPs were used to define linkage groups with *SeparateChromosomes2* module of *lepMap3*, with default parameters (lodLimit = 10). Additional markers were added to linkage groups with *JoinSingles2All* with lodLimit = 8 and lodDifference = 2. The markers on each of the chromosomes were ordered and recombination distances were measured with *OrderMarkers2* tool in the *lepMap3* software [31].

To locate the position of the sex locus in the genetic map we added a ‘sexSNP' to the filtered set of SNPs prior to genetic mapping. The sexSNP was heterozygous in all males and homozygous for the same allele in all females, corresponding to male heterogamety of *G. biloba* reported by the previous studies [29,30]. *OrderMarkers2* placed the sexSNP next to gene *evm.chr2.642* in the non-recombining block of markers on chromosome 2 located at 72.53 and 68.34 cM in the male and female maps, respectively. Consistent with the location of the sexSNP in the genetic map, the same block of markers contained the genes with Y-specific SNPs and was also the hotspot for reciprocal best blast hits for sex-linked genes identified in the previous studies (table 5; electronic supplementary material, table S3). We reasoned that this block of genes corresponds to, or contains *G. biloba* SDR.

Reconstruction of the Y-linked gametologues for X-linked genes was based on the presence of Y-specific SNPs that were detected in four of the genes in the non-recombining block of markers on chromosome 2. Such Y-SNPs represent SNP positions where all males have the alternative allele in heterozygous configuration and all females have the homozygous reference allele, in the genes transcribed in both sexes. The number of SNPs and Y-SNPs per gene in the non-recombining block of genes on chromosome 2 are listed in the electronic supplementary material, table S3. The reads mapping to the X-linked genes with Y-SNPs were extracted from bam files with a combination of *samtools* and *grep* programs. The resulting gene-specific sam files were processed with the custom-written *filterSAMbyVCF* v. 0.1 program (available at https://filtersambyvcf.sourceforge.io) to separate the sequence reads containing the Y-SNPs, along with their paired reads into a separate Y.sam file. The Y.sam file was subsequently used to call Y-consensus with *ProSeq3* v. 3.994 [63].

### (e) Evolutionary genetic analyses

Tajima's relative rates test [36], as implemented in *MEGA* v. 10.1.5 software [64], was conducted separately for three codon positions in each of the four genes with X- and Y-linked sequences available for analysis (table 3). Pairwise sequence divergence at silent and non-silent sites (table 2), as well as the phylogeny reconstruction (figure 3) were conducted with *MEGA* software [64]. The outgroup sequences from *Picea glauca* were found with NCBI *blastn*. The tripartite alignments including X-linked, Y-linked and the outgroup sequence for genes *evm.chr2.642* to *evm.chr2.645* were created and manually checked with *ProSeq3* [63].

## Data Availability

Newly generated transcriptome sequence data were submitted to NCBI under BioProject no. PRJNA758727. The accession numbers for individual samples are listed in the electronic supplementary material, table S1 [65].
